# Supplementary figures and images for: Exploring disparities and novel insights into metabolic and cardiovascular comorbidities among COVID-19 patients in Mexico
Source: Front Public Health. 2023 Oct 20;11:1270404. doi: 10.3389/fpubh.2023.1270404 (PMC10623435; doi:10.3389/fpubh.2023.1270404)

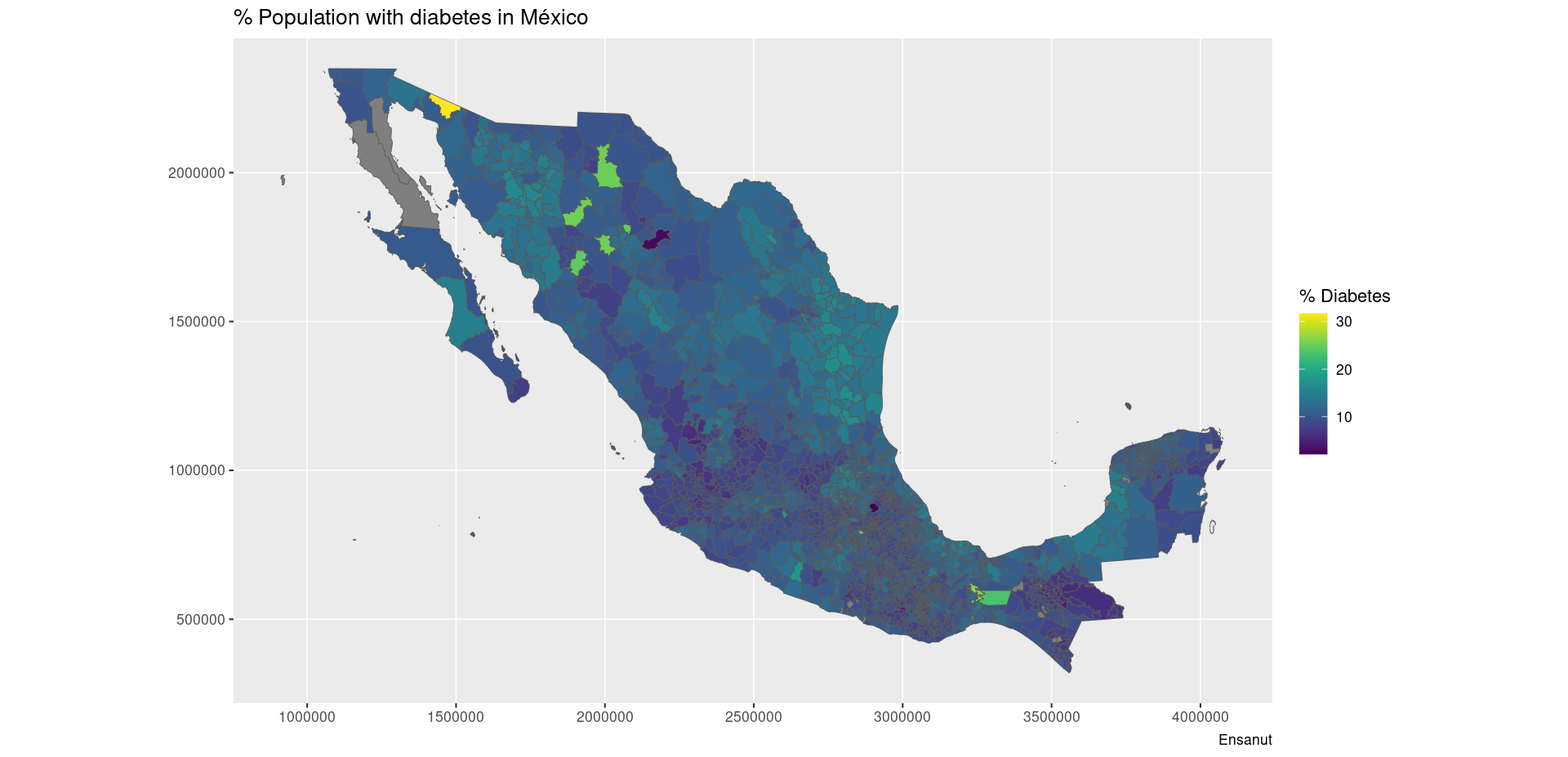

Supplement: Supplementary file 1 [file Presentation_1.zip › Mapa_diabetes.png]

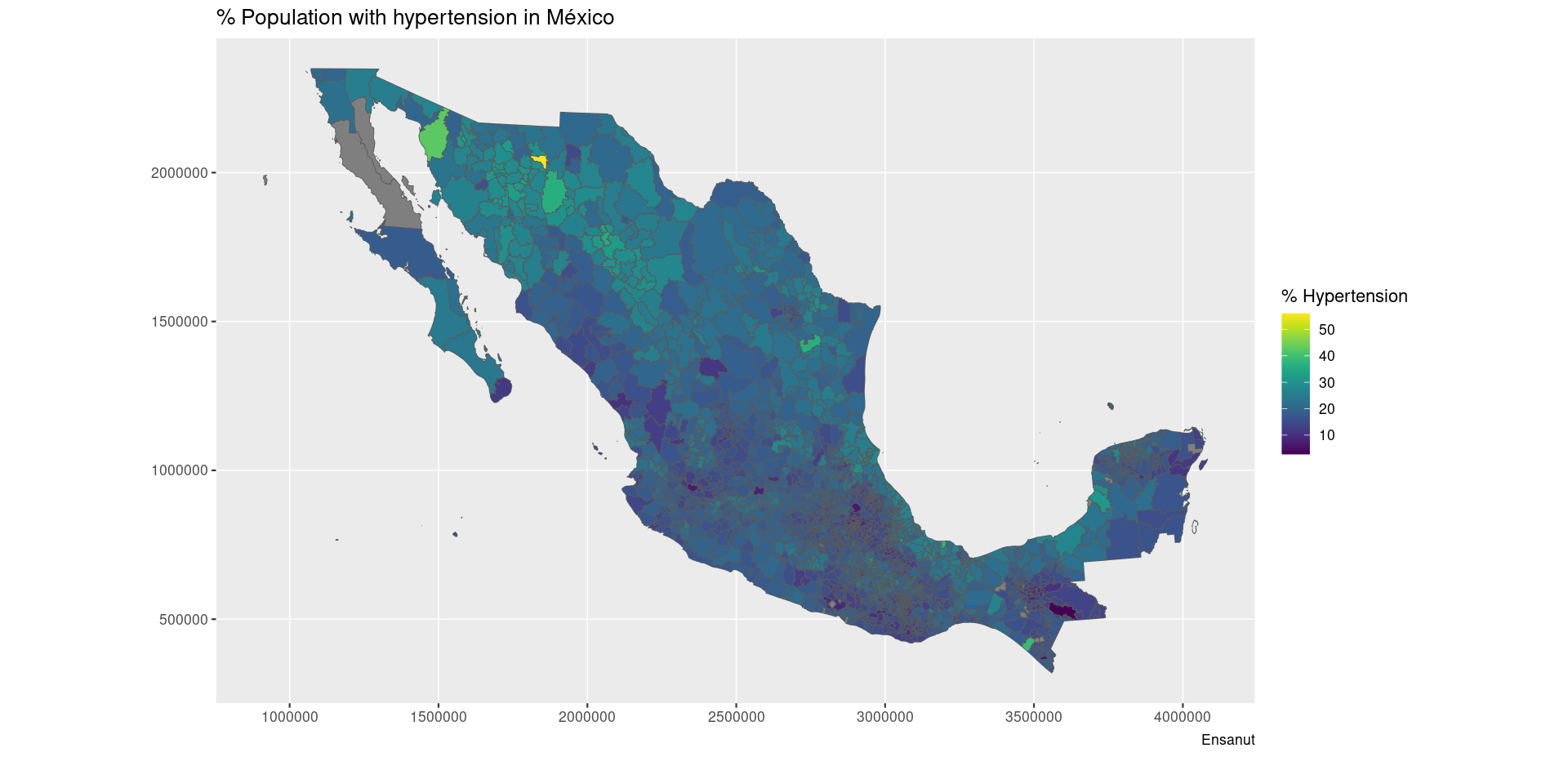

Supplement: Supplementary file 1 [file Presentation_1.zip › Mapa_hypertension.png]

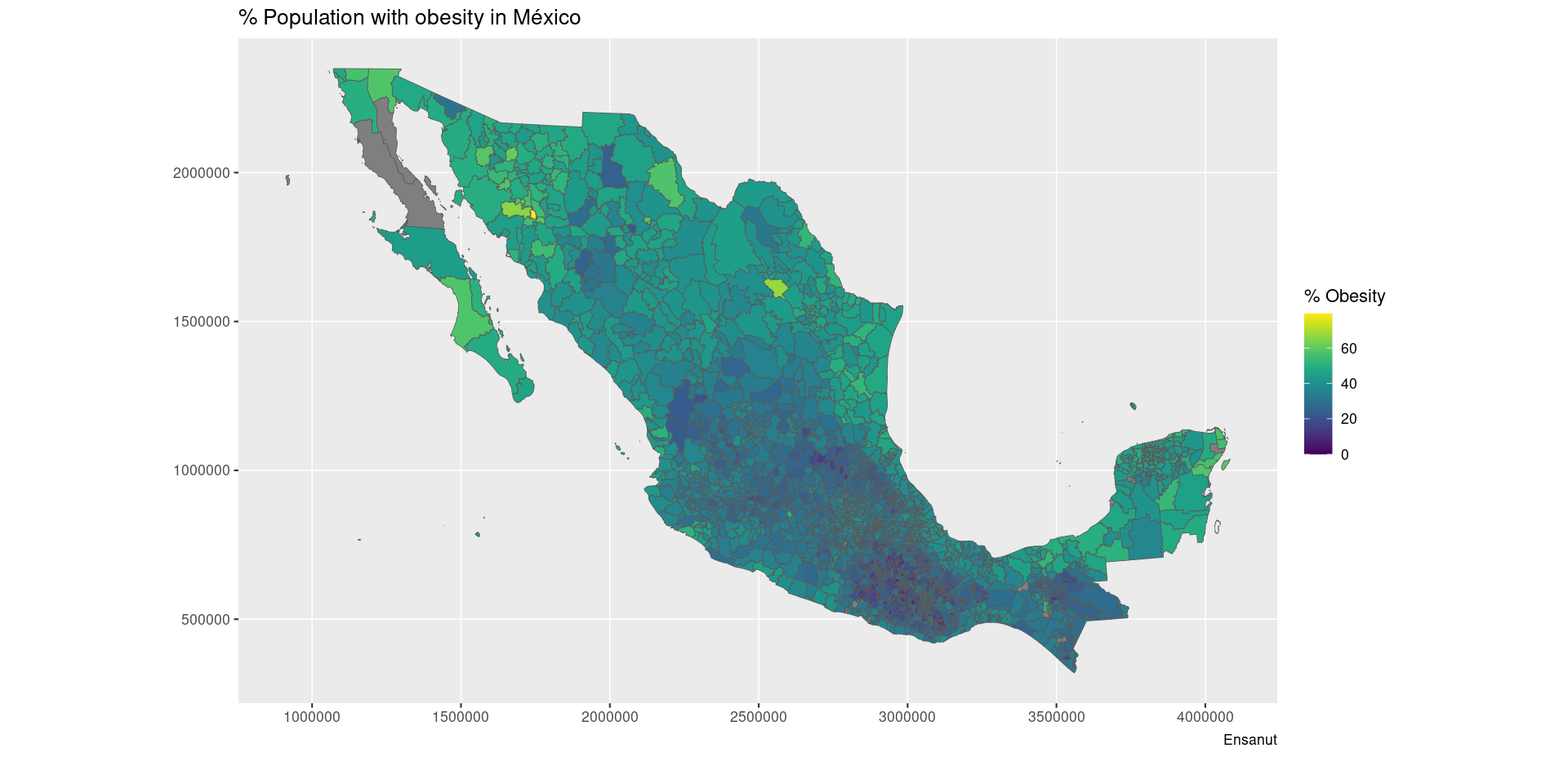

Supplement: Supplementary file 1 [file Presentation_1.zip › Mapa_obesity.png]

COVID-19 death rate

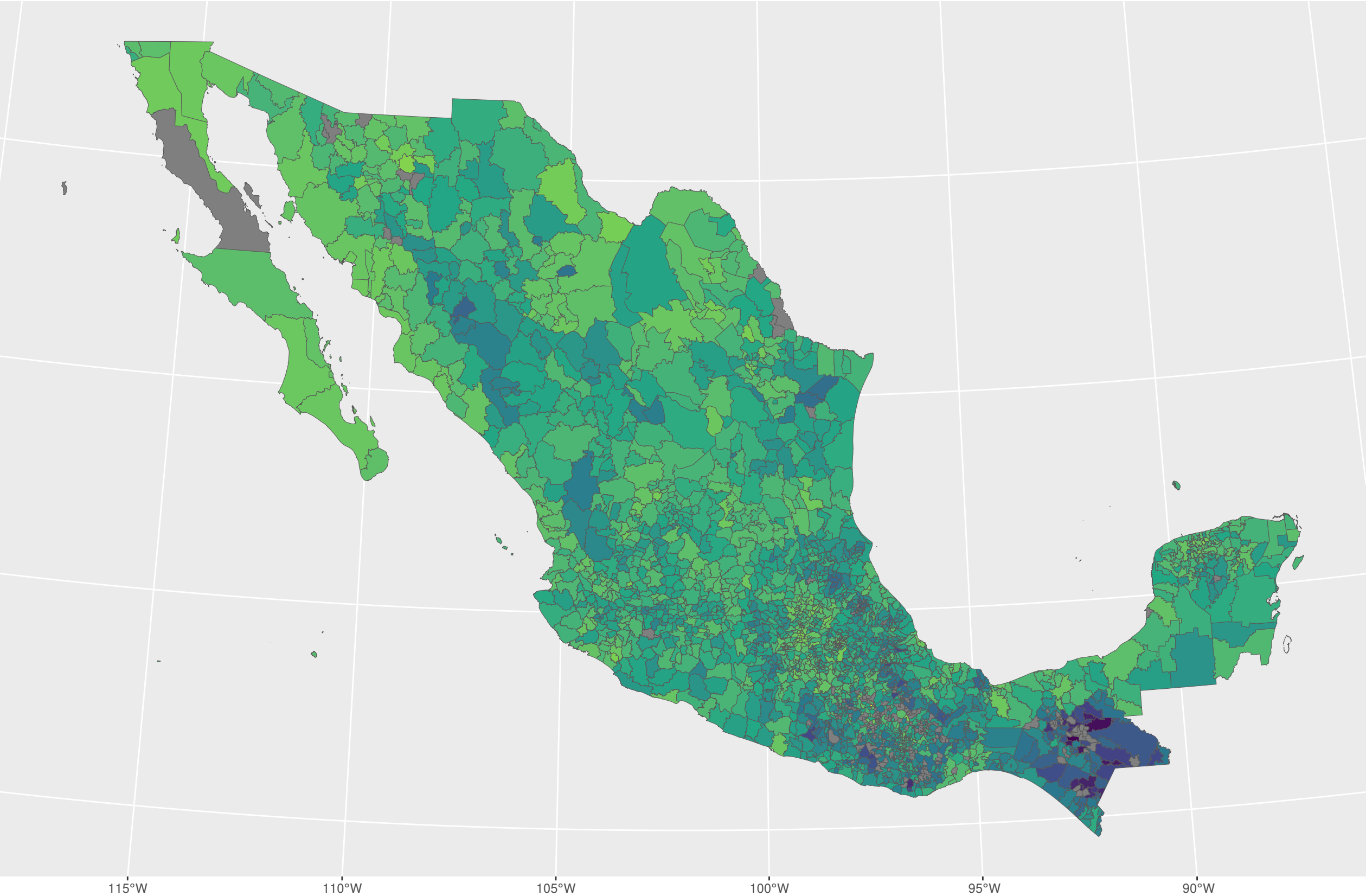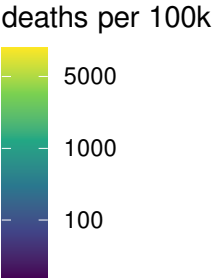

Supplement: Supplementary file 1 [file Presentation_1.zip › mapa_deathrate_log.pdf]

COVID-19 incidence

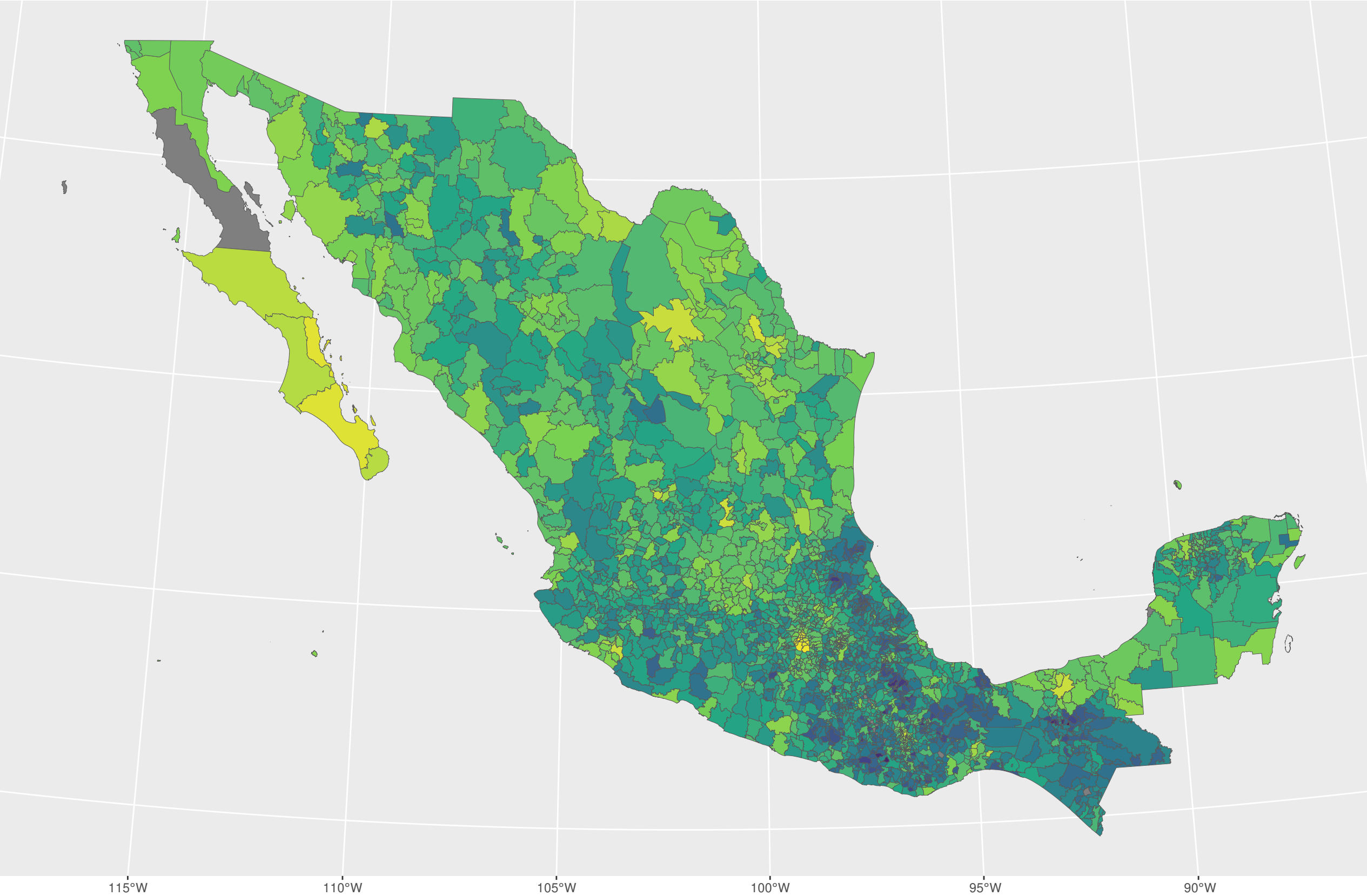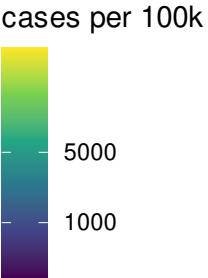

Supplement: Supplementary file 1 [file Presentation_1.zip › mapa_incidencia_log.pdf]

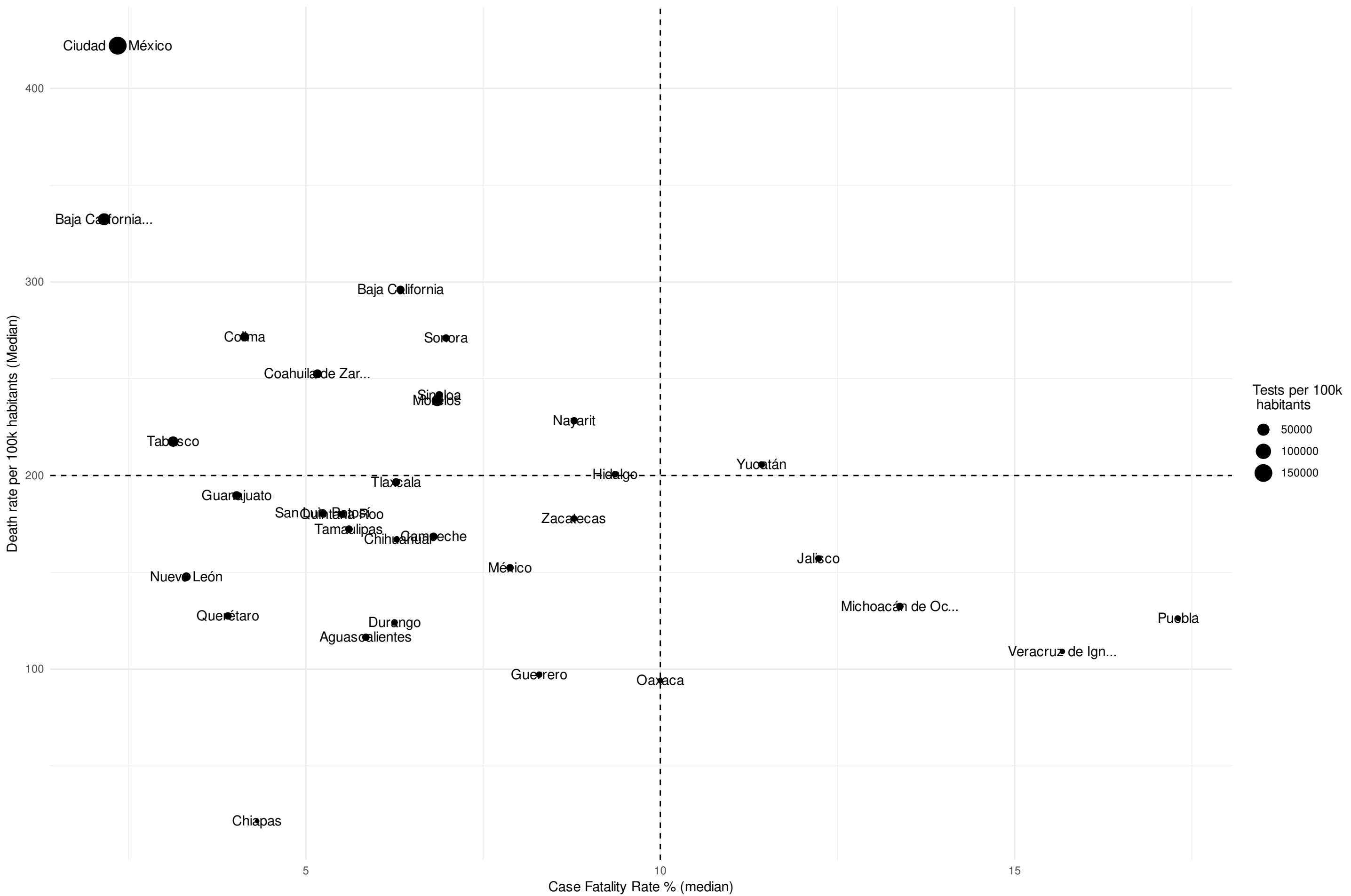

Supplement: Supplementary file 2 [file Presentation_2.pdf]
